# Evaluation of paclitaxel-loaded polymeric nanoparticles in 3D tumor model: impact of tumor stroma on penetration and efficacy

**DOI:** 10.1007/s13346-023-01310-1

**Published:** 2023-02-28

**Authors:** Dwi L. Priwitaningrum, Kunal Pednekar, Alexandros V. Gabriël, Aida A. Varela-Moreira, Severine Le Gac, Ivo Vellekoop, Gert Storm, Wim E. Hennink, Jai Prakash

**Affiliations:** 1 Engineered Therapeutics, Department of Advanced Organ Bioengineering and Therapeutics, TechMed Centre, Faculty of Science and Technology, University of Twente, Drienerlolaan 5, 7500AE Enschede, The Netherlands; 2 Department of Pharmaceutics, Faculty of Pharmacy, Universitas Sumatera Utara, Medan, Indonesia; 3 Department of Pharmaceutics, Utrecht Institute for Pharmaceutical Sciences, Faculty of Science, Utrecht University, Utrecht, The Netherlands; 4 Applied Microfluidics for BioEngineering Research, Faculty of Electrical Engineering, Mathematics and Computer Science, MESA+ Institute for Nanotechnology, TechMed Centre, University of Twente, Enschede, The Netherlands; 5 Biomedical Photonic Imaging, Faculty of Science and Technology, University of Twente, Enschede, The Netherlands

**Keywords:** Nanomedicine, Polymeric nanoparticles, Tumor stroma, Tumor penetration, 3D spheroids, Therapeutic efficacy

## Abstract

Since tumor stroma poses as a barrier to achieve efficacy of nanomedicines, it is essential to evaluate nano-chemotherapeutics in stroma-mimicking 3D models that reliably predict their behavior regarding these hurdles limiting efficacy. In this study, we evaluated the effect of paclitaxel-loaded polymeric micelles (PTX-PMCs) and polymeric nanoparticles (PTX-PNPs) in a tumor stroma–mimicking 3D in vitro model. PTX-PMCs (77 nm) based on a amphiphilic block copolymer of mPEG-b-p(HPMAm-Bz) and PTX-PNPs (159 nm) based on poly(lactic-co-glycolic acid) were prepared, which had an encapsulation efficiency (EE%) of 81 ± 15% and 45 ± 8%, respectively. 3D homospheroids of mouse 4T1 breast cancer cells and heterospheroids of NIH3T3 fibroblasts and 4T1 (5:1 ratio) were prepared and characterized with high content two-photon microscopy and immunostaining. Data showed an induction of epithelial-mesenchymal transition (α-SMA) in both homo- and heterospheroids, while ECM (collagen) deposition only in heterospheroids. Two-photon imaging revealed that both fluorescently labeled PMCs and PNPs penetrated into the core of homospheroids and only PMCs penetrated into heterospheroids. Furthermore, PTX-PMCs, PTX-PNPs, and free PTX induced cytotoxicity in tumor cells and fibroblasts grown as monolayer, but these effects were substantially reduced in 3D models, in particular in heterospheroids. Gene expression analysis showed that heterospheroids had a significant increase of drug resistance markers (Bcl2, Abgc2) compared to 2D or 3D monocultures. Altogether, this study shows that the efficacy of nanotherapeutics is challenged by stroma-induced poor penetration and development of resistant phenotype. Therefore, this tumor stroma–mimicking 3D model can provide an excellent platform to study penetration and effects of nanotherapeutics before in vivo studies.

**Graphical Abstract:**

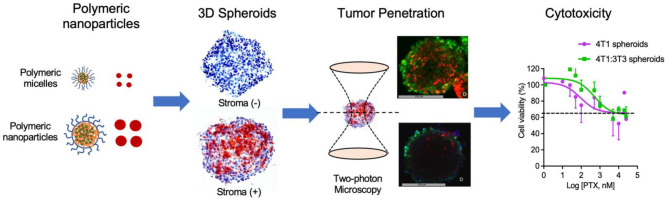

**Supplementary Information:**

The online version contains supplementary material available at 10.1007/s13346-023-01310-1.

## Introduction

In the last decades, nanoparticles have been extensively used to deliver chemotherapeutic agents to the tumor microenvironment in order to enhance therapeutic efficacy and reduce adverse effects of the loaded drugs [1, 2]. Many of the developed nanomedicines are evaluated for their cellular uptake and anti-tumor effects in two-dimensional (2D) tumor cell cultures in vitro, followed by in vivo evaluation in mouse tumor models. Studies have shown that tumor cells acquire a resistant and proliferative phenotype when grown in the 3D form which make them difficult to kill, that is not shown in 2D culture [3, 4]. Furthermore, in recent years, we and others have shown that most solid tumors develop abundant tumor stroma, fibrotic tissue in the surrounding of tumor cells composed of extracellular matrix (ECM) and cancer-associated fibroblasts (CAFs). The tumor stroma acts as a physical barrier for the penetration of nanomedicine, leading to poor intratumoral distribution [5–11]. Moreover, studies have demonstrated that CAFs within the breast tumor stroma interact with tumor cells and stimulate their proliferation and confer resistance to chemotherapy by secreting various growth factors and cytokines [12–14]. Therefore, many chemotherapeutics containing nanomedicines that show cytotoxic effects in 2D cell cultures could be overestimated for its therapeutic efficacy in vivo due to missing the key characteristics of the presence of tumor stroma in 2D models [3, 15]. It makes such a gap in the preclinical evaluation of nanomedicines to allow their translation from in vitro results to in vivo animal models to human clinical trials.

Thus, it is important to consider the complex interaction of the tumor microenvironment with tumor cells while developing models for nanomedicine evaluation, especially in in vitro ones, in order to obtain reliable data for bridging the gap between 2D in vitro and the in vivo situation. 3D in vitro models are frequently used as different cells can be included to mimic the tumor microenvironment [16]. In a previous study, we developed a microarray platform based on 3D stroma–containing heterospheroids to mimic tumor-stroma interaction [17]. Furthermore, we demonstrated that introduction of tumor stroma components in the spheroids hampered nanoparticle penetration. The physicochemical properties of the nanoparticles such as size, charge, type of polymer, type of encapsulated drug, and way of drug loading appeared to be critical factors determining the efficacy of the nanomedicines in the 3D models.

Highly hydrophobic drugs, a typical characteristic of many chemotherapeutic drugs, are incorporated into nanoparticles by solubilization in polymeric micelles based on amphiphilic block copolymers [18–20] or by physical encapsulation into polymeric nanoparticles such as poly(lactic-co-glycolic acid) (PLGA)–based nanoparticles [21–26]. In the current study, we aimed to study the effect of tumor stroma on the efficacy of paclitaxel (PTX)–loaded nanoparticles in the form of polymeric micelles and nanoparticles. PTX was physically loaded in both mPEG-b-p(HPMAm-Bz) polymeric micelles and PLGA polymeric nanoparticles. Both types of PTX nanoparticulate delivery systems were characterized for their physicochemical properties and studied for their uptake by tumor cells as well as penetration capability in a suitable 3D in vitro model. We chose 3D breast tumor spheroid model because the breast tumor contains an abundant amount of stroma/CAFs (about 50% of the tumor mass), as shown by us earlier in an analysis on human tissue microarray [18]. Also we developed mouse breast tumor spheroid models with or without stroma and well characterized them for stroma content, thus providing a high confidence of accuracy and reproducibility for the current study. Homospheroids (single culture cell type) and heterospheroids (co-culture of tumor cells and fibroblasts) were prepared and subsequently characterized for cellular organization using two-photon microscopy and for the expression of tumor stromal biomarkers. In order to investigate the effect of stroma on nanoparticle penetration, mono- and heterospheroids were incubated with fluorescently labeled polymeric micelles (size around 70 nm) and PLGA nanoparticles (size around 150 nm) for up to 48 h and then examined for their intra-spheroidal distribution. Finally, the cytotoxicity of PTX-loaded polymeric micelles (PTX-PMCs) and PTX-loaded PLGA nanoparticles (PTX-PNPs) were studied in mono- and heterospheroids.

## Materials and methods

### Materials

High glucose Dulbecco’s Modified Eagle Medium (DMEM) 4.5 g/l with L-glutamine, RPMI-1640 without L-glutamine, and L-glutamine was purchased from PAA/GE Healthcare (Eindhoven, the Netherlands). Dulbecco’s Phosphate Buffered Saline (DPBS) without calcium and magnesium was purchased from Lonza Benelux BV (Breda, the Netherlands). Trypsin–EDTA 0.5%, fetal bovine serum (FBS), Cell Tracker™ Green CMFDA (5-chloromethyluorescein diacetate), Cell Tracker™ Blue CMAC (7-amino-4-chloromethylcoumarin), and Cell Tracker™ Orange CMTMR (5-(and-6)-(((4-chloromethyl)benzoyl)amino) tetramethylrhodamine) were purchased from Life Technologies (Bleiswijk, the Netherlands). Penicillin/streptomycin, polyvinyl alcohol (PVA, Mw 30,000–70,000), paclitaxel, resazurin sodium salt used for alamar blue solution, Pluronic®F-127, and hematoxylin were purchased from Sigma-Aldrich (Zwijndrecht, the Netherlands). PTX dissolved in DMSO (Thermo Scientific) as a 1 mM stock solution was stored at − 80 °C in aliquots of 50 µl. Uncapped PLGA (lactide/glycolide molar ratio 50:50, IV = 0.4 dl/g, molecular weight of 44,000 Da) was obtained from Corbion (Gorinchem, the Netherlands). mPEG_2000_-PLGA_44000_ (lactide/glycolide molar ratio 50:50) was synthesized by ring opening polymerization [22] and characterized as described previously [26]. Ethyl acetate was obtained from VWR chemicals (Amsterdam, the Netherlands). Tetrahudrofuran (THF) was a product of Biosolve Ltd. (Valkenswaard, the Netherlands). Regenerated cellulose membrane filters of 0.45 μm were purchased from Phenomenex (Utrecht, the Netherlands). 3-Amino-9-ethyl-carbazole (AEC Red) was from Invitrogen (Breda, the Netherlands). VectaMount™ Permanent Mounting Medium and Aquatex® aqueous mounting medium were purchased from Vector Laboratories (Peterborough, UK) and Millipore (Billerica, MA, USA), respectively. CellTiter-Glo® 3D cell viability assay obtained from Promega (Madison WI, USA). Firefly luciferase assay kit was from Biotium (Fremont, CA, USA). Milli-Q water was obtained using Millipore Advantage A10 (Billerica, MA, USA).

### Preparation of nanoparticles

#### PLGA nanoparticle preparation (PNPs)

PLGA nanoparticles were prepared by an emulsion solvent evaporation method as described previously with minor adjustments [21, 26]. In brief, a mixture of mPEG_2000_-PLGA_44000_ and PLGA (3:7 w/w) was dissolved in 2 ml ethyl acetate; the total polymer concentration was 2.5% w/v. Next, 100 μl of DMSO (for empty nanoparticles) or 100 μl of PTX solution (20 mg/ml) in DMSO (for PTX-loaded NPs) was added to the polymer solution while vortexing, and subsequently sonicating for 30 s at 20% power output. Subsequently, the solution was added dropwise to 2 ml of 2% PVA (w/v) under constant vortexing at maximum speed. The emulsification was performed in an ice-bath using a microtip probe sonicator (Branson Sonifier 250, Branson Ultrasonics Corporation, Danbury, Connecticut, USA) for 2 min at 5% power output. The formed o/w emulsion (2 ml) was transferred into 45 ml of 0.3% PVA (w/v) and stirred overnight at RT to evaporate ethyl acetate and solidify the emulsified droplets. The formed particles were isolated by centrifugation for 60 min at 16,000 rpm (38,000 g) (Rotor SS-34, Sorvall RC-5C Plus, Kendro Lab, Asheville, North Carolina, USA). Finally, the particles were washed with 30 ml of PBS and thereafter with water, and finally lyophilized. The resulting dry nanoparticles were suspended in Milli-Q® water (100 µg/ml) and their average size and size distribution were measured by dynamic light scattering (DLS) using a Nano ZS Zetasizer (Malvern Panalytical, Malvern, UK) at 20 °C. Nanoparticles were also suspended in 20 mM HEPES, and injected into a disposable capillary cell DTS 1070 for zeta potential measurement and analyzed using Smoluchowski approach [27].

The paclitaxel loading and encapsulation efficiencies were determined using UHPLC (Thermo Scientific™ UltiMate™ 3000 BioRS System) as reported previously by the company and adapted from Alvi et al. [28]. In brief, 10 mg of lyophilized PTX-loaded NPs was dissolved in 250 μl of DMSO and incubated at 50 °C and agitated at 400 rpm for 30 min. Next, 750 μl of 50% ACN in Milli-Q® water with 0.1% TFA was added. The mixture was incubated at 50 °C in for an additional 6 h at 400 rpm to allow dissolution of the drug and precipitation of the polymer which was separated from the dissolved drug by centrifugation for 20 min at 20,000 g. The supernatant with the solubilized drug was collected and subsequently 20 μl was injected and analyzed with UPLC (Thermo Scientific™ UltiMate™ 3000 BioRS System). The UPLC system was equipped with an Acquity UPLC C18 Column (1.7 µm, 2.1 mm × 50 mm) and an ultraviolet detector (Diode Array, Dionex, Thermo Scientific) at 227 nm. The mobile phase consisted of Milli-Q® water (solvent A) and acetonitrile/methanol (40:60) (solvent B). A linear gradient was applied from 50 to 90% solvent B in 4 min at a flow rate of 0.45 ml/min. The drug content was calculated using a calibration curve obtained by injection of samples with known concentrations of paclitaxel (0.2 to 1500 µg/ml). The encapsulation efficiency is defined as the amount of paclitaxel entrapped divided by the feeding paclitaxel × 100%. Loading efficiency is defined as the encapsulated amount of paclitaxel divided by dry weight of loaded nanoparticles × 100%.

In vitro drug release from PTX-loaded nanoparticles was determined in PBS (pH 7.4, NaCl 0.14 M, KCl 0.03 M, Na_2_HPO_4_ 0.08 M, NaH_2_PO_4_.H_2_0 0.01 M) as reported previously with minor adjustments. In short, PTX-loaded nanoparticles (corresponding to 2 mg of PTX) were suspended in 5 ml of PBS (composition given above) and incubated at 37 °C under mild agitation. At different time points, the tube was centrifuged at 22,000 g for 1 h at 4 °C [21]. Next, 500 µl of supernatant mixed with 500 µl of acetonitrile was analyzed by HPLC as described above.

#### Polymeric micelles preparation (PMC)

Polymeric micelles were prepared using a block copolymer of mPEG-b-p(HPMAm-Bz) (M_n_ of 22 kDa, mPEG of 5 kDa) synthesized by free radical polymerization via a macroinitiator route [29]. In brief, 500 μl of a block copolymer of mPEG-b-p(HPMAm-Bz) solution in THF (60 mg/ml) was mixed with 500 μl PTX solution in THF (20 mg/ml) (for PTX-PMC) or 500 μl of THF (for non-loaded polymeric micelles) [20, 30]. Subsequently, the organic phase was added dropped into 1 ml water while vigorously stirring for about 1 min to yield micelles. THF was evaporated by stirring the micellar dispersion overnight. Subsequently, the volume was adjusted to 1 ml with 10 × concentrated HEPES-buffered saline (HBS) (200 mM HEPES, 1500 mM sodium chloride, pH 7.4) to yield micellar dispersions in HBS and subsequently filtered through a 0.45 μm membrane to remove precipitated/non encapsulated drug. PMCs were characterized for their average size and size distribution using a Nano ZS Zetasizer and for zeta potential measurement using a disposable capillary cell DTS 1070 (see the “PLGA nanoparticle preparation (PNPs)” section). The Z-average size was analyzed using cumulants and CONTIN approaches and reported using number percentage value.

The encapsulation efficiency and drug loading were determined by analyzing micelle samples dissolved in acetonitrile using HPLC as reported previously [31]. In short, one volume of PMC was added to nine volumes of acetonitrile (ACN) to disrupt the micelles and solubilize the loaded drug. Encapsulation efficiency and drug loading were calculated as follows: encapsulation efficiency % = weight of loaded drug/weight of feeding drug × 100% and drug loading % = weight of loaded drug/weight of (loaded drug + polymer) × 100%.

#### Cyanine-3 labeling

Non-loaded PLGA NPs were labeled with cyanine-3 using carbodiimide chemistry via EDC/NHS activation yielding Cy3-PNP [26]. In short, 50 μl of a solution of 40 mM EDC/NHS in MES Buffer (0.5 M, 975 mg MES dissolved in 10 ml Milli-Q® water, adjusted to pH 6.3 with Na_2_CO_3_ 2.5 M) was added to 200 μl loaded nanoparticle suspension (25 mg/ml) and allowed to react for 45 min at room temperature. Next, the formed activated nanoparticles were resuspended in 200 μl of PBS and reacted with 10 μl of Cy3-amine in DMF (10 mg/ml) for 2 h at room temperature. The resulting Cy3-labeled PNPs (Cy3-PNPs) were purified using an Amicon® column by washing with PBS thrice, resuspended in PBS (25 mg/ml), and stored at 4 °C. Cy-3-labeled polymeric micelles (Cy3-PMCs) were prepared using mPEG-bp(HPMAm-Bz) with covalently linked Cy3 as described previously [30]. Subsequently, Cy3-PMCs were prepared similarly to the empty polymeric micelles as described in the “Polymeric micelles preparation (PMC)” section.

Furthermore, the resulting Cy3-PNP and Cy3-PNP dispersions were used for uptake and penetration study in 2D and 3D culture, respectively (the “In vitro uptake of Cy-3-labeled PLGA NPs and polymeric micelles (Cy3-PNP and Cy3-PMC) by 4T1 cells as monolayer culture” and “Penetration of nanoparticles” sections).

### Cell culture

Mouse 4T1 breast cancer cells and murine NIH3T3 fibroblasts were obtained from American Type Culture Collection (ATCC, Rockville, MD). 4T1 cells were cultured in RPMI 1640 medium, supplemented with 10% fetal bovine serum (FBS), 2 mM L-glutamine, and antibiotics (50 U/ml Penicillin and 50 μg/ml streptomycin). NIH3T3 cells were cultured in Dulbecco’s modified Eagle’s medium (DMEM) supplemented with 10% FBS, 2 mM L-glutamine, and antibiotics (50 U/ml Penicillin and 50 μg/ml streptomycin). The cells were grown in cell culture–treated 75 cm^2^ flasks in a humidified incubator at 37 °C with 5% CO_2_ were passaged regularly using 0.05% trypsin–EDTA in PBS to detach the cells.

### In vitro uptake of Cy-3-labeled PLGA NPs and polymeric micelles (Cy3-PNP and Cy3-PMC) by 4T1 cells as monolayer culture

4T1 cells were seeded in a 24-well plate (1 × 10^4^ cells/well; medium given in the “Cell culture” section) and incubated for 24 h in a humidified incubator at 37 °C with 5% CO_2_. Next, 5 μl of Cy3-labeled PNPs or PMC dispersed in PBS (final concentration of Cy3 was 5 μM; see also the “Cyanine-3 labeling” section) was added. After 2- and 18-h incubation, the cells were evaluated using fluorescence imaging. The nuclei were visualized by NucBlue™ Fixed Cell ReadyProbes™ Reagent–containing DAPI. The uptake of Cy3-labeled nanoparticles was imaged 20 min after nuclei staining at room temperature. Cy3 label $({\lambda }_{\mathrm{Ex}/\mathrm{Em}}555/570\mathrm{ nm})$ and nuclei were detected through Texas Red and DAPI filter, respectively, using EVOS® FL Color Imaging System (Life Technologies).

### 3D-spheroid formation and characterization

#### 3D-spheroid array formation

Microwell array–containing Petri dishes were obtained as previously described by hot embossing microwell arrays (108 wells of 200 μm depth, 400 μm diameter) using a home-made set-up [32]. The dishes were incubated overnight with 1% w/v Pluronic F-127 in PBS to render the surface protein repellant and minimize cell adhesion. 4T1 homospheroids were generated by seeding 2 × 10^6^ 4T1 cells, while a seeding total of 2 × 10^6^ cells of 3T3 and 4T1 cells with a ratio of 5:1 for generating heterospheroids in the embossed-microwell array Petri dish and centrifuged at 2500 rpm (1100 g) for 5 min to force disposition of the cells in the microwells [18](. The culture medium was aspirated, the excess of cells removed, and the dishes were washed thoroughly with PBS twice. Fresh 1 ml DMEM medium was subsequently added and the dishes placed back in the incubator at 37 °C with 5% CO_2_ for spheroid formation for 48 h. The spheroids were used for the experiments afterwards.

#### Characterization of spheroids

##### Two-photon microscope imaging

In order to characterize the 3D heterospheroids cellular arrangement using two-photon microscope, 4T1 cells and 3T3 cells were incubated for 1 h at 37 °C with 15 μM CellTracker™ Green and 15 μM CellTracker™ Blue in culture media, respectively, to label the cells prior to seeding in microwell array Petri dish. Next, the medium was removed and the cells were washed twice with PBS. Labeled cells were proceeded further for spheroid formation as described in the “3D-spheroid array formation” section. The resulting spheroids were imaged and scanned every 15 μm depth using two-photon microscope system developed by Biomedical Photonic Imaging (BMPI) group of University of Twente, Enschede, the Netherlands [33]. CellTracker™ Green fluorescence showing 4T1 tumor cells and CellTracker™ Blue fluorescence showing 3T3 cells were excited at different wavelength of 905 and 765 nm, respectively. The wavelengths were optimal for the two-photon excitation peaks for the fluorophores. Images retrieved were processed with NIH ImageJ software.

##### Immunostaining of CAF biomarkers

Spheroid characterization was performed for expression of the CAF (cancer-associated fibroblast) biomarkers by immunostaining. Spheroids grown in microwells for 48 h were washed with PBS and subsequently embedded in Cryomatrix™, cut into 8-μm-thick sections and processed for immunostaining. Cryosections were fixed with acetone at room temperature (RT) for 15 min, rehydrated in PBS, and incubated with either mouse anti-α-SMA (Sigma-Aldrich, 1:400) or goat anti-collagen type I (Southern Biotech, 1:100) in PBS for 1 h at RT. Subsequently, sections were washed in PBS again and incubated with secondary antibody—horseradish peroxidase (HRP)–labeled rabbit anti-mouse IgG (DAKO, 1:100) or HRP-labeled goat anti-rabbit IgG (DAKO, 1:100) in PBS for 1 h. Sections were rewashed with PBS and finally incubated with a tertiary antibody—HRP-labeled goat anti-rabbit IgG (DAKO, 1:100) or HRP-labeled rabbit anti-goat IgG (DAKO, 1:100) in PBS for 1 h. AEC (3-amino-9-ethyl-carbazole) in Milli-Q water was applied for 20 min to develop peroxidase activity and resulting in red staining. Samples were subsequently counterstained with hematoxylin to visualize cell nuclei, washed in running tap water for 5 min, and mounted with Aquatex®. Imaging was performed using Nanozoomer-RS.

## Penetration of nanoparticles

The nanoparticle penetration study was performed using 48-h-old spheroids that were isolated and placed in a 96-well plate that was pre-incubated with 1% (w/v) Pluronic F-127, as described previously [32]. Homospheroids of 4T1 tumor cells and heterospheroids of 3T3 and 4T1 cells (5:1) were incubated with 1.25 mg/ml of Cy3-labeled polymeric micelles (Cy3-PMC) and Cy3-labeled PLGA nanoparticles (Cy3-PNP) suspended in serum-free DMEM medium. Spheroids were incubated in a humidified incubator at 37 °C with 5% CO_2_. After incubation with the nanoparticles for 24 h, the spheroids were washed with PBS and subsequently mounted in 3 ml serum-free DMEM medium (composition given in the “Cell culture” section) in a 35-mm Petri dish. The spheroids were centered and imaged using a two-photon microscope system to observe the penetration of the nanoparticles into spheroids. CellTracker™ Green fluorescence showing 4T1 tumor cells was excited at a wavelength of 905 nm. CellTracker™ Blue fluorescence showing 3T3 cells was excited at a wavelength of 765 nm. Those wavelengths were optimal for the two-photon excitation peaks for the fluorophores. Red fluorescence representing PLGA NPs or polymeric micelles conjugated to Cy3 dye was excited at a two-photon excitation peak wavelength of 1020 nm. Subsequently, the nanoparticle penetration was quantified digitally using NIH ImageJ software.

## In vitro effect of PTX-loaded PLGA NPs and polymeric micelles on cell viability in 2D culture and 3D stroma–containing tumor model

### 2D cell viability assay

Cell viability assay was performed using Alamar Blue assay to investigate the dose response of 4T1 cells, 3T3 cells, or 3T3/4T1 cell mixture viability towards the PTX-loaded nanoparticles in in vitro 2D culture. Cells were seeded at a density of 2500 cells per well in a 96-well plate. 4T1 cells were cultured in RPMI medium, and 3T3 cells and 3T3/4T1 cell mixture were cultured in DMEM medium (composition given in the “Cell culture” section) for 24 h. Subsequently, PTX-PMC, PTX-PNPs, and free PTX (PTX concentration ranging from 25 nM to 25 μM) were added and the cells were incubated for 48 h in a humidified incubator at 37 °C with 5% CO_2_. PTX was dissolved in DMSO and administered in corresponding cell medium with final concentration of DMSO was less than 0.1%v/v. Next, 110 μl of Alamar Blue solution (440 μM resazurin salt in PBS, diluted 1:10 v/v in RPMI medium) was added to the wells and the cells were incubated for an additional 4 h at 37 °C. Fluorescence of reduced resazurin was subsequently measured using Victor3 1420 Multilabel Counter (Perkin Elmer, Waltham, MA, USA) at excitation/emission wavelengths of 560/590 nm. The cell viability ratio was calculated with the following equation: cell viability (%) = (the fluorescence of treated cells in medium—fluorescence of medium only)/(fluorescence of non-treated cells in medium—fluorescence of medium only) × 100%.

### 3D cell viability assay

The effect of PTX-loaded nanoparticles on cell viability of 3D culture of 4T1 as homospheroid and with 3T3 cells as heterospheroid was investigated using CellTiter-Glo® 3D cell viability assay. Homospheroids of 4T1 and heterospheroids of 3T3/4T1 (5:1) were obtained as described in the “3D-spheroid array formation” section. Spheroids were incubated with PTX-PMC, PTX-PNPs, or free PTX (PTX concentration ranging from 25 nM to 25 μM), after which 48-h incubation at 37 °C, 5% CO_2_ followed. Free PTX was dissolved in DMSO and administered in corresponding cell medium with final concentration of DMSO was less than 0.1%v/v. Next, the treated spheroid-containing well-plates and CellTiter-Glo® 3D (CT-Glo 3D) reagents were equilibrated for ca. 30 min at RT prior to addition of the 100 µL CT-Glo 3D reagent into 100 µL of culture medium present in each well. Subsequently, the treated spheroids and CT-Glo 3D reagent–containing well-plates were mixed by placing them on an orbital shaking platform for 5 min. Thereafter, the plates were incubated at RT for 25 min in order to stabilize the luminescent signal which was recorded using Infinite®200 PRO plate reader (Tecan, Männedorf, Switzerland), at an integration time of 1000 ms and 5 ms settle time. Spheroid growth was monitored by imaging the spheroids directly and at 48 h after treatments using an EVOS™ M5000 imaging system (Thermofisher Scientific, Washington, USA) at 4 × magnification. The spheroid diameters were measured using ImageJ software.

## Results and discussion

### Preparation of nanoparticles and cyanine-3 labeling

**Fig. 1:**
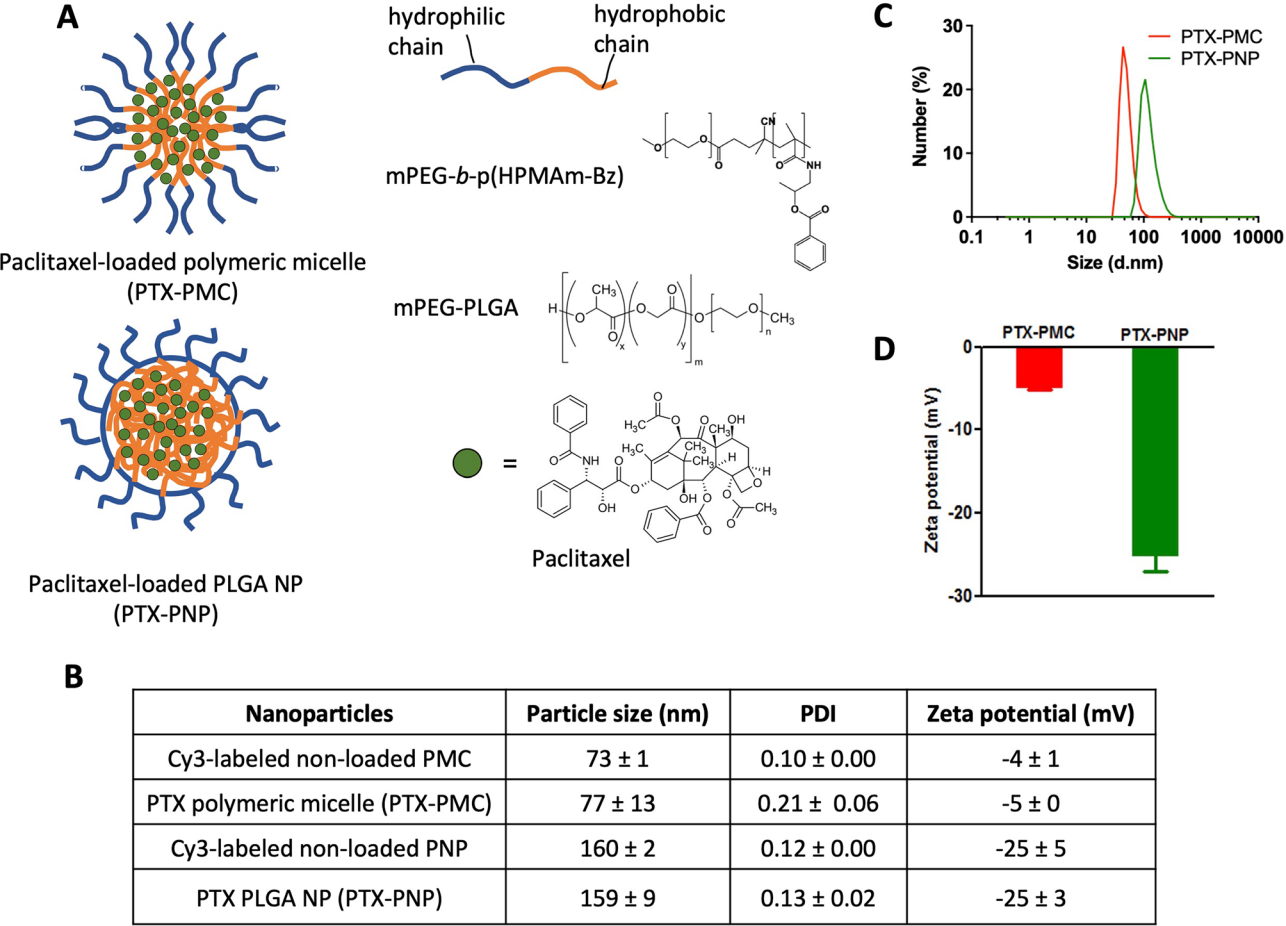
PTX-loaded micelles and PLGA nanoparticles. **A** Schematic illustration of PTX-loaded micelles and PLGA NPs. **B** Characteristics of non-loaded and loaded micelles and PLGA NPs. **C** Size distribution of PTX-loaded micelles (PTX-PMC) and PLGA NPs (PTX-PNP). **D** Zeta potential of PTX-PMC and PTX-PNP in 20 mM HEPES buffer. Data are shown in mean ± SD

mPEG-b-p(HPMAm-Bz)-based polymeric micelles (PMC) and PLGA nanoparticles (PNPs) were prepared using a solvent evaporation method and an oil-in-water emulsification method, respectively. Schematic illustration of PTX-loaded micelles and PLGA NPs can be seen in Fig. 1A. PTX, one of the most effective chemotherapeutics used to treat different types of cancers, such as lung, ovarian, and breast [34–36], was loaded into PMC exploiting π-π stacking interactions resulting in PTX-PMC with of diameter 77 ± 13 nm and polydispersity index (PDI) of 0.23 ± 0.06 (Fig. 1B, C). These results are in good agreement with the previous study using the same polymer and the same procedure used for preparing the micelles which had size in the range of 60 to 80 nm (19). PTX was also encapsulated into PNP resulting into PTX-PNP of an average diameter of 159 ± 9.2 nm with a PDI of 0.07 ± 0.02 (Fig. 1B, C). The zeta potential for the PTX-loaded PLGA NPs was more negative than that of polymeric micelles (− 25.3 ± 3.16 versus − 5.0 ± 0.31 mV) (Fig. 1B, D) which can be ascribed to the terminal carboxylate group (COO–) of uncapped PLGA, in line of the previous studies [25, 37]. Encapsulation and loading efficiency of PTX-PMC were 81 ± 15 and 23 ± 3%, respectively, which is in good agreement with previous findings [30, 31]. PTX-PNP had encapsulation efficiency of 45 ± 8% and loading efficiency of 1.4 ± 0.9%.

Non-loaded PMCs and PNPs covalently labeled with the fluorescent dye to Cy3 were prepared for cellular uptake studies using 4T1 cells and penetration studies in 3D breast tumor model in vitro. The mean sizes of Cy3-labeled non-loaded nanoparticles were about the same as that the PTX-loaded nanoparticles (73 and 160 nm; 77 and 159 nm, respectively).

### In vitro cellular uptake of Cy3-PMC and Cy3-PNPs by 4T1 cells in a monolayer

**Fig. 2:**
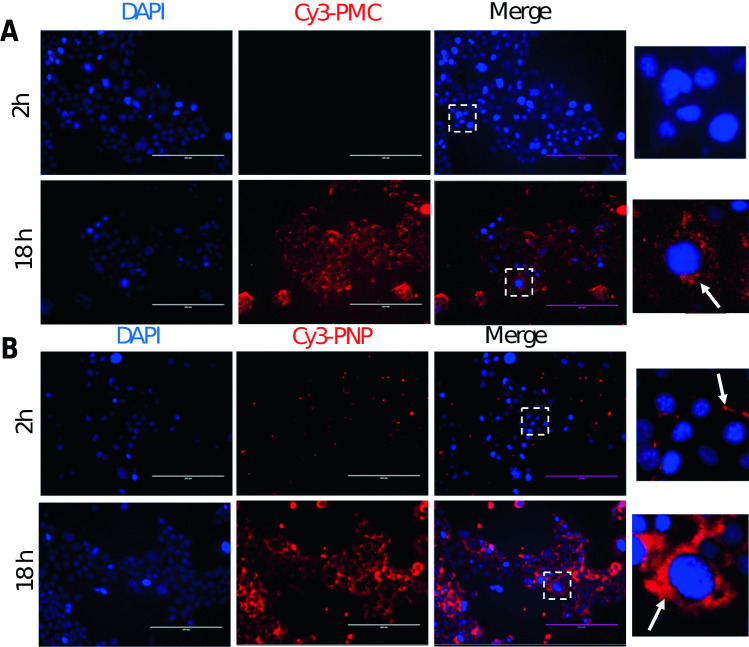
Uptake of Cy3-labeled micelles and PLGA nanoparticles by 4T1 tumor cells. **A** Representative fluorescence microscopic images showing cellular uptake of Cy3-labeled micelles and PLGA NPs, Cy3-PMC (**A**), and Cy3-PNP (**B**) by 4T1 cells after early (2 h) and late time points (18 h). Cy3-staining is shown as red color. The nuclei were stained with DAPI (blue). (right, inset) Enlarged pictures of cells showing the accumulation of the Cy3-nanocarriers accumulating at the cell membrane (2 h) and around the nuclei (18 h). Scale bar: 200 μm

The uptake of both Cy3-PMC and Cy3-PNP by 4T1 cells in the form of a monolayer was examined at early (2 h) and late time point (18 h). As shown in Fig. 2, a weak red fluorescence signal of Cy3-PMC and Cy3-PNP was observed at the cellular membranes at 2 h in a dotted pattern. However, at 18 h, a strong fluorescence signal was observed next to the nuclei, especially the polymeric micelles that were smaller than the PNP particles (77 and 159 nm, respectively). This is in line with literature that smaller nanoparticles normally show higher cellular uptake [38–40]. The data demonstrate that both Cy3-PMCs and Cy3-PNPs are taken up by 4T1 cells.

### 3D spheroid formation and characterization using two-photon microscopy

**Fig. 3:**
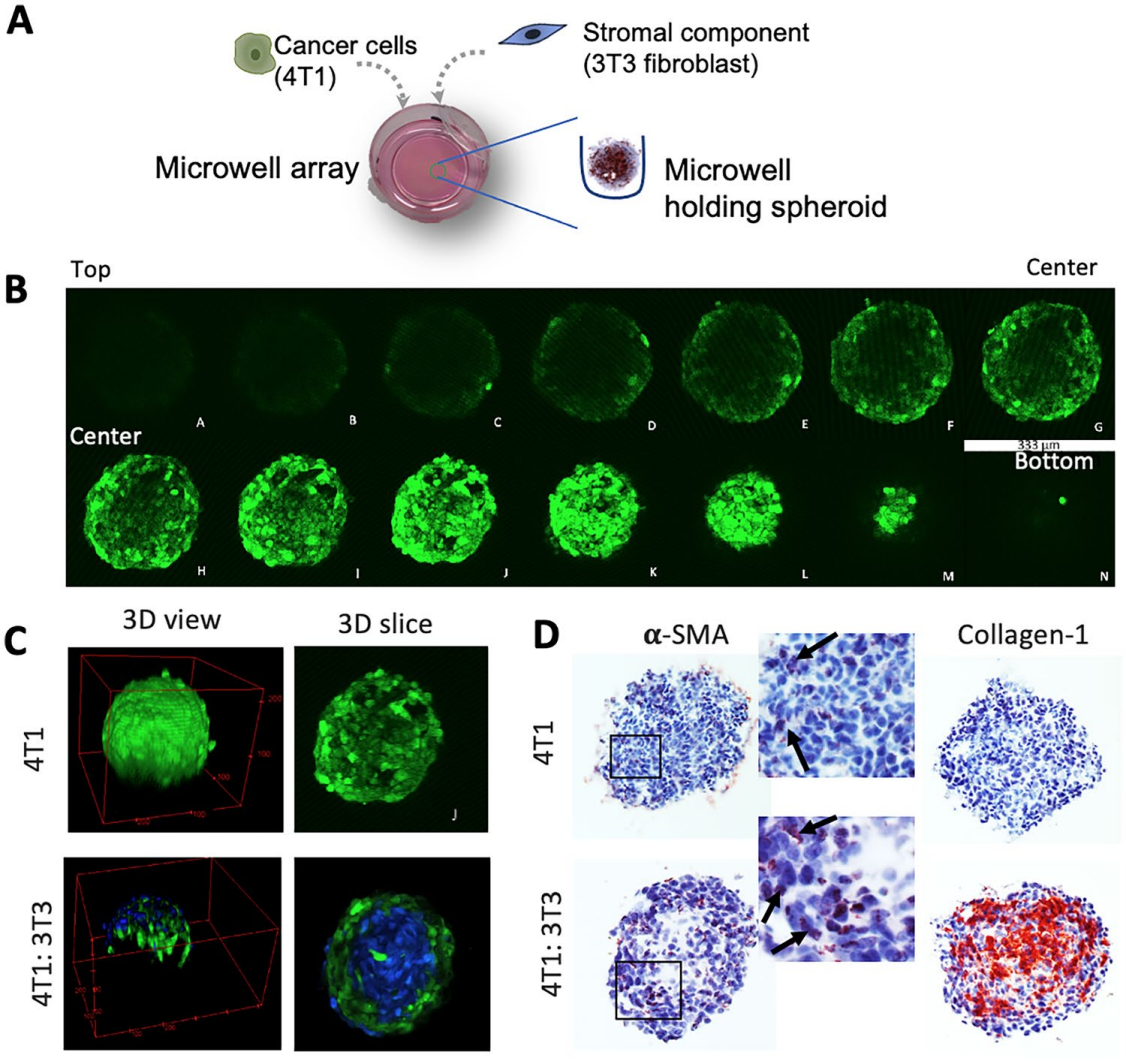
Spheroid characterization using two-photon microscopy and immunostaining. **A** Schematic diagram shows the procedure of generating 3D spheroids in microwell array. **B** *Z*-axis scanned images of 4T1 homospheroid (4T1 cells labeled with CellTracker^TM^Green) using two-photon microscope. Each slice shows the gap of 15 µm. **C** 3D view of the reconstructed a homospheroid and a heterospheroid and 3D slice of spheroids. 4T1: green; 3T3: blue. **D** Immunohistochemical images of 4T1 homospheroid and heterospheroid after α-SMA and collagen staining. Zoomed images show the α-SMA staining in both spheroid types

CAFs play a crucial role in breast tumor by interacting with tumor cells and stimulating their proliferation and conferring resistance to chemotherapy [13–15]. Also, CAFs produce abundant ECM which acts as a physical barrier for drug and nanoparticle penetration. To mimic the 3D microenvironment and tumor stroma, homospheroids composed of 4T1 cells only and heterospheroids composed of 4T1 mouse tumor cells and NIH3T3 mouse fibroblasts (1:5 ratio) were prepared (Fig. 3A), as reported previously [17].

These spheroids were characterized for their integrity and cellular organization using two-photon microscopy. Earlier we used histological analysis and confocal microscopy to characterize them. However, histological analysis has some drawbacks including time consumption, damage of the tissue, and insufficient control on depth from the surface as well as that fluorescently labeled nanoparticles may lose their signal during the immunostaining procedure [41, 42]. In addition, confocal microscopy allows live imaging but has limited light penetration of only about 100 μm depth. Subsequently, homospheroids containing green CellTracker™-labeled 4T1 cells were scanned with two-photon microscope 48 h after seeding into the microwells. As shown in Fig. 3B, the spheroids were scanned from bottom to top and individual cells could be clearly visualized. Using these orthoslice images, cross sections of each axis were imaged, allowing to reconstruct the 3D spheroids (Fig. 3C). Figure 3C shows the 3D view and a 3D slice of the scan of 4T1 homospheroids and heterospheroids. It can be seen that homospheroids are quite homogeneous in organization of the 4T1 cells, whereas in heterospheroids, 3T3 fibroblasts (CellTracker^TM^Blue) orient themselves towards the center and 4T1 cells (cellTracker^TM^Green) organize themselves around the 3T3 fibroblasts. These data are in line with our previous findings [17]. Since fibroblasts are contractile and have strong cell-to-cell interactions, they tend to attach each other and form a dense and compact structure as compared to homospheroids composed of 4T1 cells only. Altogether, the two-photon microscopic analysis allows for high content imaging for determining shape, cellular organization, and cell–cell interactions in complex 3D structures.

The two-photon imaging data were confirmed with histological analysis by cryosectioning the spheroids and immunohistochemical staining for fibroblasts biomarker (α-SMA) and ECM protein (collagen-1) (Fig. 3D). Interestingly, it was found that 4T1 tumor cells, which are non-mesenchymal cells, expressed α-SMA which indicates that these cells undergo epithelial-mesenchymal transition (EMT) in 3D culture. Earlier 4T1 tumor cells showed to undergo EMT in vivo [43] which confirms that our 3D spheroids mimic the in vivo phenotype of tumor cells. Furthermore, in heterospheroids, expression of α-SMA was much evident in both the fibroblasts and tumor cells, as spheroid edges which are mainly composed of tumor cells were also strongly positive (Fig. 3D, zoomed image). The data thus indicate that 4T1 tumor cells likely undergo EMT and attain mesenchymal phenotype due to cross-talk with cancer-associated fibroblasts (CAFs). Our data are in line with a previous study which showed that CAFs induce EMT in tumor cells by secreting growth factors such as TGF-β [44]. Furthermore, 3D heterospheroids strongly expressed collagen which was not present in fibroblasts-lacking homospheroids (Fig. 3D). Although tumor cells underwent EMT in homospheroids, they do not have capacity to produce collagen which is the key function of CAFs. Overall, the data demonstrate that the tumor-stroma interaction takes place in the 3D heterospheroid culture which is an important phenomenon for inducing tumor cell invasion, development of resistance against chemotherapeutic drugs, and forming a barrier against these therapeutics [11].

### Penetration of nanoparticles into 3D spheroids

**Fig. 4:**
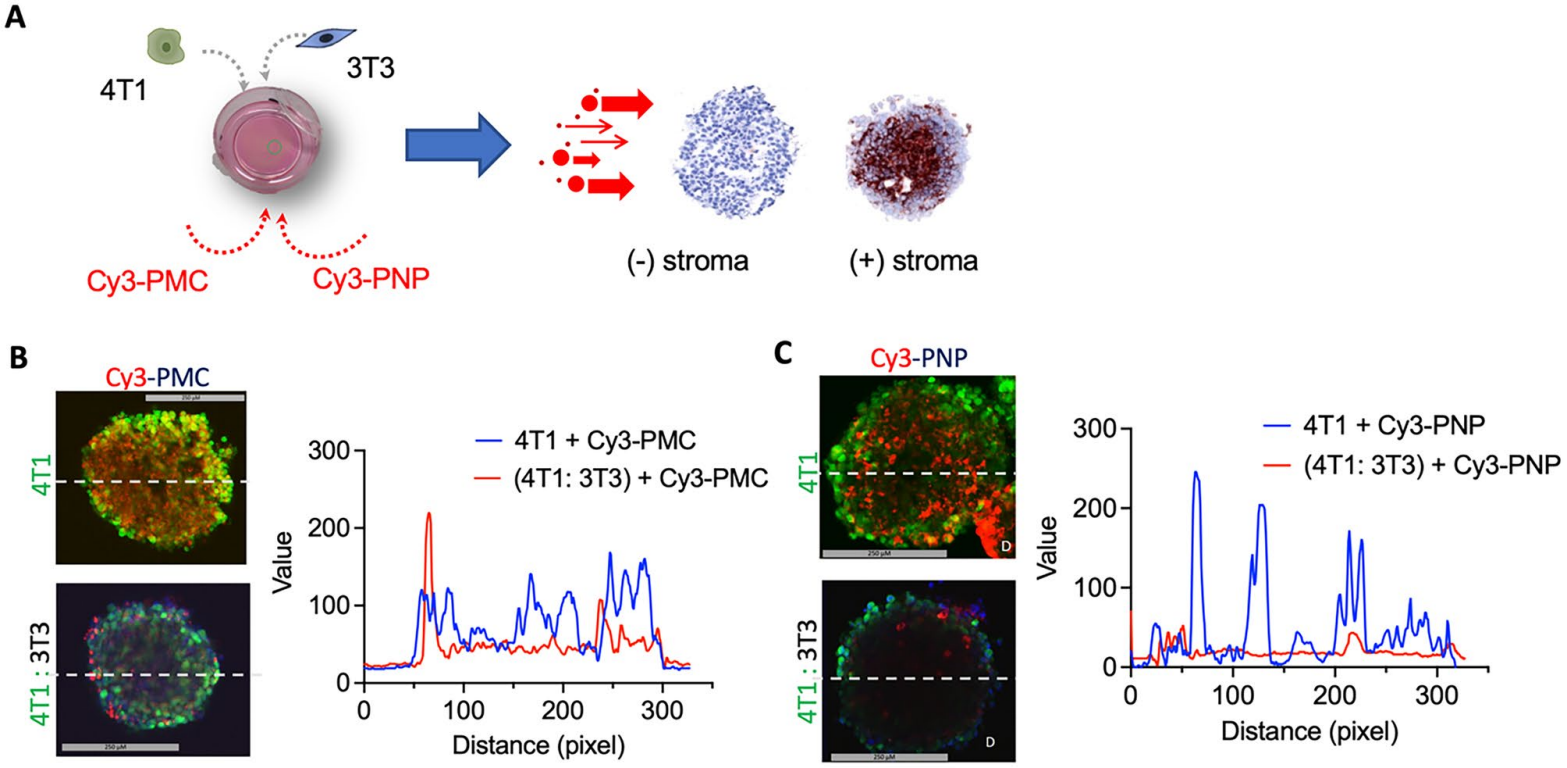
Penetration of Cy3-labeled nanoparticles in 3D spheroids using two-photon microscopy. **A** Schematics showing incubation of Cy3-PMC and Cy3-PNP with 3D 4T1 homospheroids and 4T1:3T3 heterospheroids. 4T1 cells were stained with CellTracker^TM^Green and 3T3 were stained with CellTracker.^TM^Blue. **B**, **C** Two-photon microscopic images show the penetration of Cy3-PMC and Cy3-PNP, respectively and plots with the intensity profile of the red signal across the spheroids (shown with the dotted lines)

To study the penetration of Cy3-PMC and Cy3-PNP into 3D spheroids, 4T1 homospheroids and heterospheroids were incubated for 24 h with different nanoparticles and then scanned with two-photon microscope (Fig. 4A). As shown in Fig. 4B and C, a cross-section image at the middle of the spheroids was taken of each condition and analyzed for the signal from nanoparticles (red color). Representative intensity profile plots show that the signal (blue line) is distributed throughout the spheroid demonstrating that both Cy3-PMC and Cy3-PNP penetrated into the 4T1 homospheroids and even reached the core (Fig. 4B and C). However, in heterospheroids, the signals from both Cy3-PMC and Cy3-PNP were mainly seen at the edges of the spheroids. The low penetration of micelles is likely due to denser collagen permeating throughout the heterospheroids which acts as a physical barrier for the penetration. Interestingly, Cy3-PMC showed a better penetration compared to Cy3-PNP in heterospheroids which is likely attributed to their smaller size (70 vs 150 nm, respectively). Positively charged NP (even small ones) will likely only stick to the cells in the outer rim of the spheroids and barely penetrate into spheroids [45].

### In vitro effect of PTX-loaded PMC and PNPs in 2D cell cultures and 3D stroma–containing tumor model

**Fig. 5:**
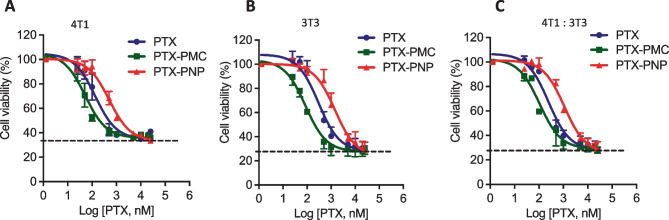
Efficacy study of PTX formulations in in vitro 2D culture. Dose response curve of free PTX, PTX-loaded micelles, and PTX-loaded PLGA NPs on cell viability of 4T1 cell (**A**), 3T3 cell (**B**), and co-culture of 3t3 and 4T1 (5:1) (**C**) in 2D monolayer cell culture. Cell viability was examined by using Alamar Blue

The in vitro cytotoxicity effect of free PTX, PTX-loaded polymeric micelles (PTX-PMC), and PTX-loaded PLGA nanoparticles (PTX-PNP) on 2D monolayer culture of 4T1 breast tumor cells, 3T3 fibroblasts, and co-culture of 3T3:4T1 (5:1) was studied using Alamar blue cell viability assay. The dose response curves show that free PTX and both PTX-PMC and PTX-PNP killed tumor cells (IC_50_ (mean + SE) of 136 + 1.3, 52.3 + 1.2, 506 + 1.2 nM, respectively) as well as 3T3 fibroblasts (IC_50_ of 299 + 1.4, 86 + 1.3, 1438 + 1.35 nM, respectively) individually and as a 4T1 + 3T3 co-culture (IC_50_ of 245 + 1.25, 106 + 1.3, and 1108 + 1.2 nM, respectively) in a concentration-dependent manner after 48 h of incubation (Fig. 5A–C).

Interestingly, PTX-PMC showed a higher cytotoxicity than both PTX and PTX-PNP, while PTX-PNPs were the least effective. The higher cytotoxicity of the PTX-PMC formulation as compared to free PTX is in line with previous findings [30]. It is also in good agreement with the results of study (the “Penetration of nanoparticles into 3D spheroids” section) in which it was shown that polymeric micelles had better spheroid penetration. Likely, the PTX-loaded micelles are internalized by cells and subsequently release their content intracellularly to trigger cytotoxic effects [31, 46]. Indeed, Sheybanifard et al. [46] showed a release of 60% in 48 h while PTX-PNP showed only 22% release of the loading in the same timeframe (Supplementary Fig. 1). This low PTX-PNP release is in line with other studies of PTX-loaded PLGA nanoparticles [21, 47, 48].

**Fig. 6:**
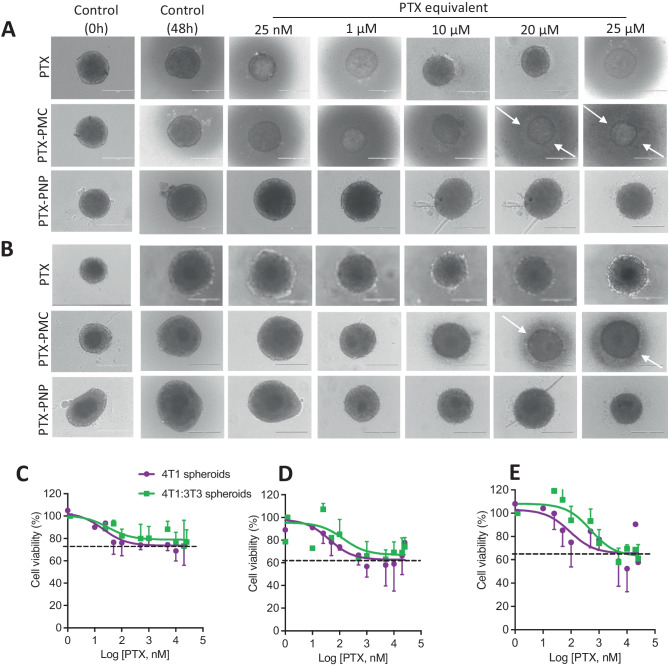
Efficacy study of PTX and PTX-loaded nanoparticles in 3D spheroids. Brightfield images showing the effect of PTX, PTX-loaded polymeric micelles (PTX-PMC), and PTX-loaded polymeric nanoparticles (PTX-PNP) on homospheroids (**A**) and heterospheroids (**B**). **C**–**E** % cell viability of 4T1 homospheroid and heterospheroid of 4T1:3T3 (1:5) after 48 h incubation with PTX formulations (**C**: free PTX, **D**: PTX-PMC, **E**: PTX-PNP). Cell debris was more prominent in PTX-PMC formulation (pointed by white arrow). Scale bar: 400 μm

As a next step, the effect of PTX-loaded nanoparticles on cell viability was investigated in 3D homospheroids and stroma-containing heterospheroids. As shown in Fig. 6A and B, the stroma-free homospheroids shrunk in size after incubation with free PTX and PTX-loaded nanoparticles. Importantly, the effect of PTX in its free form and as nanoparticle formulations was much weaker on 4T1 homospheroids compared to their monolayer counterparts (Fig. 6 vs. Fig. 5). Of note, with the treatment with PTX-PMC, we observed an aura (indicated with arrows) of cell debris in the surrounding of the spheroids which is likely due to the erosion of the outer cell layers due to PTX-mediated cytotoxicity.

**Fig. 7:**
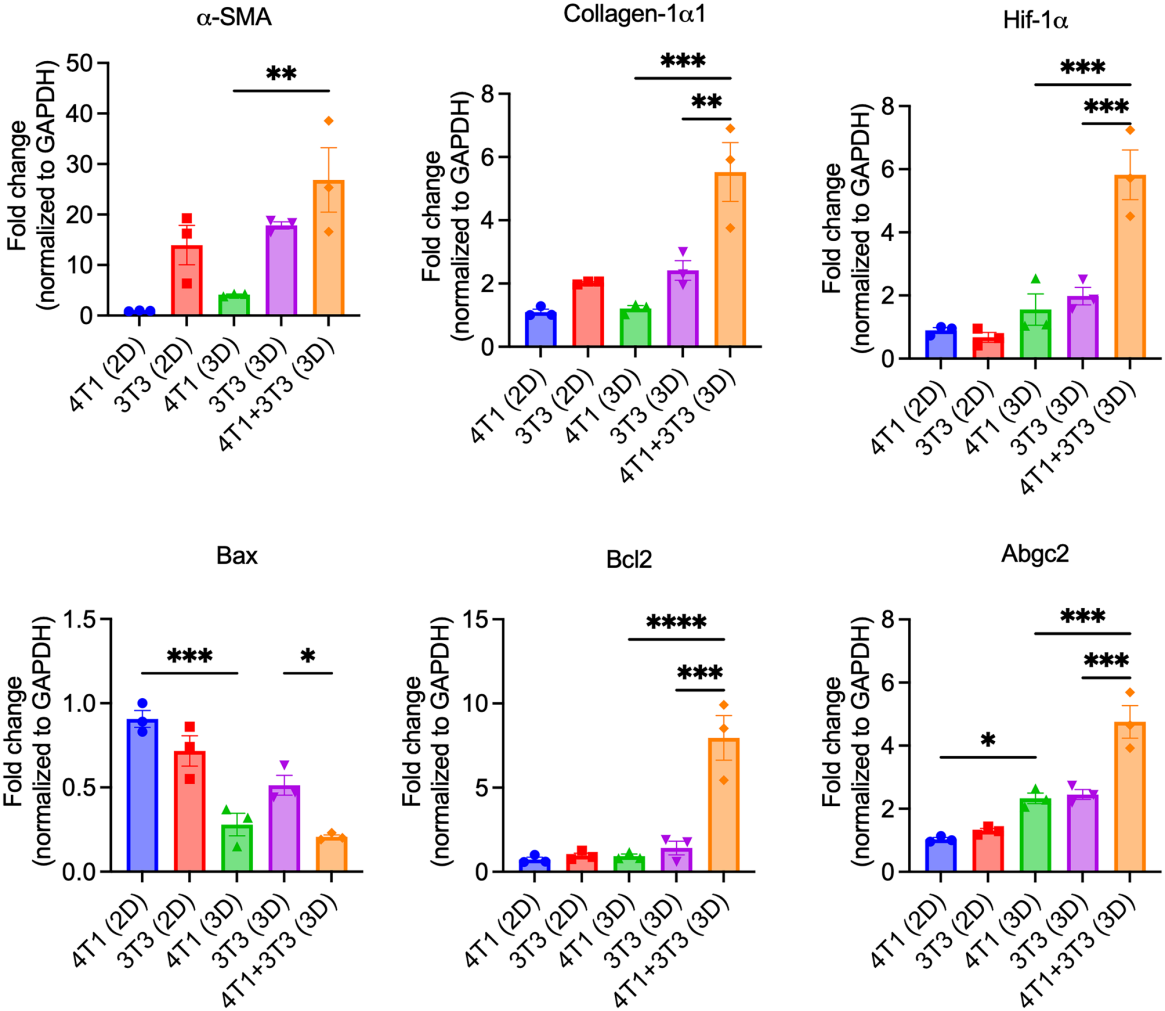
Gene expression analysis of markers related to stroma, EMT, and drug resistance. Data show mean ± SEM, *N* = 3. Ordinary one-way ANOVA analysis using GraphPad Prism ver. 9, *p < 0.05, **p < 0.01, ***p < 0.001

Importantly, PTX-loaded nanoparticles had lower inhibitory effects on the stroma-containing heterospheroids compared to the homospheroids (Fig. 6C–E). This might be attributed to the poor penetration of nanoparticles besides the attainment of drug-resistant phenotype in 3D culture (vide infra). The PTX-PMC (Fig. 6D) showed better inhibitory effects compared to free PTX (Fig. 6C) and PTX-PNP (Fig. 6E), likely due to their smaller size that allowed better penetration. PTX-PNP was the least efficacious likely due to poor penetration due to their relatively large size as compared to the PTX-PMC micelles. Furthermore, we performed gene expression analysis on the 2D cultures and 3D spheroids to examine changes that occurred due to culture in 3D and interaction with stroma (Fig. 7). Interestingly, it was found that 4T1 cells showed expression of α-SMA (mesenchymal marker), Abgc2 (ATP-binding cassette transporter responsible for resistance), and a decrease in expression of Bax (BCL2 Associated X, responsible for apoptosis) in 3D spheroids. These data indicate that the formation of 3D spheroids already turns tumor cells into more resistant phenotype due to mesenchymal transition. Furthermore, co-culture of 4T1 and 3T3 in 3D showed a significant increase in expression of stroma markers (α-SMA, collagen-1α1), hypoxia marker (HIF-1α), and drug resistance markers (Bcl2, Abgc2) and decrease in expression of Bax compared to 3D monocultures. These data convincingly demonstrate that tumor cell-stroma interactions lead to induction of EMT and hypoxia as well as drug resistance which are contributing factors for poor efficacy of PTX and nanoparticles in both 3D homospheroids and heterospheroids. It is also in accordance with other studies using co-culture of tumor cells and fibroblasts showed that increased expression of stroma marker of α-SMA and EMT feature only observed in 3D co-culture, not in 2D co-culture (49–50).

In conclusion, the present study demonstrates the significance of 3D culture model for the evaluation of nanomedicines before testing them in preclinical tumor models. Compared to 2D culture, tumor cells attain more resistant phenotype in 3D culture likely due to better cell-to-cell contact which drives them to undergo EMT. This became evident, as the current study showed that PTX had much lower cytotoxicity in 3D spheroids compared to 2D monolayer culture. Furthermore, addition of the tumor stroma component (fibroblasts and extracellular matrix (ECM)) in 3D culture caused a physical barrier for nanoparticle penetration, as shown with two-photon microscopy. Although the results showed that smaller sized micelles had a better penetration in 3D spheroids compared to PLGA nanoparticles, there was only a slight gain in effectivity when examined as PTX-loaded micelles. The development of drug resistance and poor penetration in 3D stroma–rich heterospheroids seem to be the key determining factors for the efficacy of nanomedicines. Different strategies have been explored to overcome tumor-stroma hurdles to enhance nanomedicine penetration. One of those strategies that has been intensively studied recently is targeting to CAFs or modulating the tumor stroma. There are some potential targets found for CAFs targeting, such as connective tissue growth factor (CTGF), integrin α11 and α5 (member of cell adhesion receptors that mediating cell migration, proliferation, survival, and cross-talk of tumor cell and ECM), and miRNA-199a and -214. Those targets can be inhibited by delivering their inhibitors or antibodies resulting in reduction of CAF-induced matrix barrier and thus improve nanomedicine penetration [51–59]. Anti-fibrotic drugs such as pirfenidone can also suppressed CAF-promoted tumor progression [60, 61]. Besides that, Mardhian et al. [6] conducted a nanomedicine study of an endogenous hormone with anti-fibrotic properties, relaxin-2, that showed significant inhibition of pancreatic tumor growth through reducing collagen expression. Also, approaches to tailor the properties of nanomedicines such as size, triggered release, and transcytosis tumor penetration have been also developed to encounter drug tumor penetration challenges [17, 62–64]. Furthermore, the developed nanomedicines can be used in combination therapy, either by co-delivery or sequential delivery of anti-stromal drugs and chemotherapeutics that have also been proven in recent reported studies in enhancing tumor penetration [13, 65, 66]. Furthermore, those strategies still need to be evaluated in a platform addressing key feature of tumor stroma–related penetration hurdles. Therefore, for this effort, the reported tumor stroma–mimicking 3D models may provide a platform for examining newly developed nanomedicines in stroma-rich 3D model before testing in vivo.

## Supplementary Information

Below is the link to the electronic supplementary material.

Supplementary file1 (DOCX 16 kb)

## Data Availability

Data and materials will be available on request to the corresponding author.
