# Supplementary material for: Evaluation of paclitaxel-loaded polymeric nanoparticles in 3D tumor model: impact of tumor stroma on penetration and efficacy
Source: Drug Deliv Transl Res. 2023 Feb 28;13(5):1470–83. doi: 10.1007/s13346-023-01310-1 (PMC10102101; doi:10.1007/s13346-023-01310-1)
Supplement: Supplementary file 1 — Supplementary file1 (DOCX 16 kb) [file 13346_2023_1310_MOESM1_ESM.docx]

**Supplementary Figure**

**Paclitaxel release from PLGA NPs**

**Figure S1.** Paclitaxel release from the PLGA nanoparticles in PBS pH 7.4 at 37^0^C. Data are presented as mean ± SEM (n=3)
